# Response of Different Genotypes of Faba Bean Plant to Drought Stress

**DOI:** 10.3390/ijms160510214

**Published:** 2015-05-05

**Authors:** Manzer H. Siddiqui, Mutahhar Y. Al-Khaishany, Mohammed A. Al-Qutami, Mohamed H. Al-Whaibi, Anil Grover, Hayssam M. Ali, Mona S. Al-Wahibi, Najat A. Bukhari

**Affiliations:** 1 Department of Botany and Microbiology, College of Science, King Saud University, Riyadh 2455, Saudi Arabia; E-Mails: muthr20@gmail.com (M.Y.A.-K.); maqutami@gmail.com (M.A.A.-O.); mwhaibi@ksu.edu.sa (M.H.A.-W.); hayhassan@ksu.edu.sa (H.M.A.); malwhibi@ksu.edu.sa (M.S.A.); najatab@ksu.edu.sa (N.A.B.); 2 Department of Plant Molecular Biology, University of Delhi South Campus, Benito Juarez Road, Dhaula Kuan, New Delhi 110021, India; E-Mail: anil.anilgrover@gmail.com

**Keywords:** growth parameters, proline, drought stress, *Vicia faba*, antioxidant enzymes, oxidative stress

## Abstract

Drought stress is one of the major abiotic stresses that are a threat to crop production worldwide. Drought stress impairs the plants growth and yield. Therefore, the aim of the present experiment was to select the tolerant genotype/s on the basis of moprpho-physiological and biochemical characteristics of 10 *Vicia faba* genotypes (Zafar 1, Zafar 2, Shebam, Makamora, Espan, Giza Blanka, Giza 3, C4, C5 and G853) under drought stress. We studied the effect of different levels of drought stress *i.e.*, (i) normal irrigation (ii) mild stress (iii) moderate stress, and (iv) severe stress on plant height (PH) plant^−1^, fresh weight (FW) and dry weight (DW) plant^−1^, area leaf^−1^, leaf relative water content (RWC), proline (Pro) content, total chlorophyll (Total *Chl*) content, electrolyte leakage (EL), malondialdehyde (MDA), hydrogen peroxide (H_2_O_2_) content, and activities of catalase (CAT), peroxidase (POD) and superoxide dismutase (SOD) of genotypes of faba bean. Drought stress reduced all growth parameters and Total *Chl* content of all genotypes. However, the deteriorating effect of drought stress on the growth performance of genotypes “C5” and “Zafar 1” were relatively low due to its better antioxidant enzymes activities (CAT, POD and SOD), and accumulation of Pro and Total *Chl*, and leaf RWC. In the study, genotype “C5” and “Zafar 1” were found to be relatively tolerant to drought stress and genotypes “G853” and “C4” were sensitive to drought stress.

## 1. Introduction

Water stress is considered a detrimental factor for the production of crops worldwide. Water scarcity is accentuated by other abiotic stresses as well as global warming [1]. Globally, more that 50% of the average yield of most major crops is lost due to drought stress (DS) [2]. Today, it has become a challenging task to combat drought stress worldwide. Like other environmental stresses, DS causes a series of physiological, biochemical and molecular changes in plants. Due to water stress, whole plant metabolism is dramatically affected by the over-production of reactive oxygen species (ROS) that are responsible for oxidation of multicellular components like proteins, lipids, DNA and RNA, resulting in death of cells [3]. Water deficit in plants causes inhibition of photosynthesis by altering pathway regulation by stomatal closure and decreasing flow of CO_2_ into mesophyll tissues and also by impairing the activity of ribulose-1,5-bisphosphate carboxylase/oxygenase [4,5,6]. Also, respiration, translocation, ion uptake, carbohydrates, nutrient assimilation and growth promoters are disturbed under stress [7,8]. Under stress, plants develop a defensive mechanism and cellular homeostasis by the accumulation of osmolytes (*i.e.*, proline, glycinebetaine) and proteins thereby increasing tolerance of plants to stress [9,10]. However, plant tolerance to abiotic stresses is a complex trait, involving a range of molecular, biochemical and physiological mechanisms [11]. The response of plants to stresses depends on species and genotypes, the length and severity of water deficit, and age and development stage [12].

*Vicia faba*, also known as faba bean*,* broad bean and fava bean, has a long history of cultivation. It is an important winter legume crop that is rich in protein and energy, and used in feed and food. According to FAOSTAT [13], China, Ethiopia, France, Egypt and Australia are main faba bean producing countries. It has significant value in improving the fertility of soil by its rotation cultivation with cereal crops; thereby, fixing nitrogen in symbiosis makes them excellent colonizers of low-N environments. The production of faba bean is not enough to feed the ever-growing world population. There are many biotic and abiotic factors that cause reduced yields. Also, bean plants showed a great magnitude of intraspecific variation [14,15], and molecular and physiological changes occur [7,8,16] for stress tolerance. Faba bean cultivation particularly in arid and semi-arid regions is unsuitable because this crop is not sufficiently drought and heat tolerant as it is susceptible to moisture and high temperature stresses [17]. Keeping in view the importance of this crop for humans as well as animals, the present experiment was planned to study the effect of DS on different genotypes of faba bean plants. The main objective of this experiment was to determine DS tolerant and sensitive genotypes on the basis of physio-morphological and biochemical parameters.

## 2. Results and Discussion

In this study, the performance of different genotypes was evaluated in terms of plant height (PH) plant^−1^, shoot fresh (SF) and shoot dry (SD) weight plant^−1^, area leaf^−1^, relative water content (RWC), total chlorophyll (Total *Chl*), proline (Pro) content, electrolyte leakage (EL), and content of malondialdehyde (MDA) and H_2_O_2_, and activity of catalase (CAT), peroxidase (POD) and superoxide dismutase (SOD). The effect of DS treatments on these parameters of genotypes was found to be significant (Table 1, Table 2, Table 3 and Table 4).

The data reveal that growth performance of faba bean genotypes were affected significantly, depending on the level of water deficit (Table 1). In general, drought stress affected all growth parameters (PH, SF weight and SD weight plant, leaf area) of plants of all genotypes. Genotype “C5” exhibited the highest values for PH and leaf area as compared to the other genotypes under severe drought stress. At severe drought stress, genotype “G853” showed the lowest value for all growth parameters. However, genotype “C5” proved to be the best by giving highest values for all growth characteristics among nine genotypes, and genotype “G853”, being at par with genotype “C4” for leaf area, had the lowest values for these parameters. A decrease in growth parameters may be due to the impairment of cell division, cell enlargement caused by loss of turgor, and inhibition of various growth metabolisms [18,19]. These results strengthen the findings of Ouzounidou *et al.* [20] on faba bean, Ali *et al.* [21] on faba bean; Farooq *et al.* [8] on rice and Asrar and Elhindi [22] on marigold, who reported that DS reduced plant growth characteristics. Among the cultivars, “C5” was found to be more tolerant by giving highest values for all growth characteristics in comparison to nine genotypes, and genotype “G853” was found to be sensitive to drought stress.

Under drought stress, leaf RWC plays an important role in tolerance of plants to stress by inducing osmotic adjustment due to the accumulation of osmoprotectants [12,23,24]. The maintenance of a high plant water status during stress is an important defensive mechanism to retain enough water by minimizing water loss (e.g., caused by stomatal closure, trichomes, reduced leaf area, senescence of older leaves, *etc.*) and maximizing water uptake (e.g., by increased root growth) [12]. In the present experiment, Table 2 and Table 3 depict that leaf RWC, Pro accumulation, Total *Chl* content, electrolyte leakage, and content of MDA and H_2_O_2_ of all genotypes were significantly affected by water stress. At increasing levels of drought stress, leaf RWC and Total *Chl* content decreased inversely. The differences in RWC in all genotypes could be associated with their ability of water absorption from soil. Thus, we conclude that genotype “C5” could have better ability to resist drought stress. According to Khanna-Chopra & Selote [25], under stress, the drought-resistant wheat plants exhibited better leaf water relations in terms of turgor potential and RWC as compared to sensitive genotypes. Genotype “C5”, being at par with genotype “Giza 3”, had the highest RWC under severe water stress conditions.

Pro accumulation increased with increasing levels of water stress (Table 2). The accumulation of Pro in plants reduces the toxic effects of ions on enzymes activity and also lowers the generation of free radicals formed by drought stress. Also, Pro associated with recovery resistance by serving a source of respiratory energy to the plants under stress [26]. Under severe DS, genotype “C5” gave the maximum value for Pro content, and genotype “G853”, being at par with genotype “Giza 3”, exhibited lower value for content of Pro. Also, genotype “C5”, followed by genotypes “Zafar 1” and “Zafar 2”, had the maximum value for Pro content (Table 2).

Under severe drought stress, genotypes “Zafar 1”, followed by genotypes “C5”, “Giza 3” and “Makamora”, gave the maximum value for Total *Chl* content (Table 2). This result strongly supports the findings of Ali *et al.* [21] in faba bean, and Mafakheri *et al.* [27] in chickpea genotypes. A decrease in Total *Chl* content may be due to the activity of chlorophyllase, a chlorophyll degrading enzyme [28]. Under drought stress, a low inhibition of Total *Chl* synthesis in genotypes Zafar 1’, “C5” and “Giza 3” could be associated with better light harvesting efficiency, thereby improving dry matter production (Table 1).

We observed that electrolyte leakage, and accumulation of MDA and H_2_O_2_ were found to be dependent on the severity of drought stress (Table 3). Genotype “C5”, being at par with genotype “Zafar 1” for electrolyte leakage, genotypes “Zafar 1”, “Giza Blanka” and Zafar 2 for MDA accumulation, gave the lowest values under severe condition of drought stress. Also, genotype “C5” showed the lowest content of H_2_O_2_ under water stress condition. All three oxidative stress indicators (electrolyte leakage, and accumulation of MDA and H_2_O_2_) were found to be almost lower in genotype “C5” and the highest in cultivars “G853”, “C4” and “Makmora”. These results agree with the findings of Ouzounidou *et al.* (20), Terzi and Kadioglu [29]; Ali *et al.* [21] and Quan *et al.* [30]. According to Jiang and Huang [31], accumulation of MDA affects the RWC and photosynthetic pigment of plants. Among the cultivars, “C5” had the lowest values for theses parameters. These results reveal that tolerance of genotype “C5” to drought stress could be positively related to leaf RWC and synthesis of Total *Chl* (Table 2).

In general, activity of antioxidant enzymes (CAT, POD, and SOD) were significantly increased with increasing levels of drought stress in plants of all genotypes, (Table 4). Under severe water stress conditions, genotype “Shebam 1”, being at par with genotype “C5”, gave a higher value for CAT activity. However, the highest enzymes activities were noted in genotypes “C5” for POD and SOD at severe level of water stress. Genotype “Zafar 1” followed by genotype “C5” for the activity of POD and SOD. Moreover, the magnitude of increase in these enzymes’ activity in genotypes “C5” was higher than other genotypes of faba bean under DS, except genotypes “Shebam 1” for CAT. Genotype “G853”, followed by genotype “C4”, exhibited the lowest enzyme activity under water stress. As we know, abiotic stress leads to the generation of reactive oxygen species (ROS: Superoxide anion radicals, hydroxyl radicals, H_2_O_2_, alkoxy radicals and singlet oxygen) that may react with a large variety of biomolecules—such as deoxyribonucleic acid, protein, lipids and carbohydrates—Causing lipid peroxidation linked membrane deterioration [32,33]. To overcome oxidative damage, plants develop an antioxidant system to scavenge ROS. In the present experiment, activity of antioxidant enzymes (POD, CAT and SOD) in plants of all genotypes increased under drought stress (Table 4). However, under DS, the highest enzymes activities were noted in genotypes “C5”, “Zafar 1”, and genotypes “G853” and “C4” exhibited the lowest value. Thus, it could be possible and reasonable to suggest that genotypes “C5” and “Zafar 1” were more tolerant than the other genotypes, because the maximum values for these enzymes’ activity were recorded (Table 4).

**Table 1:** Growth performance of faba bean genotypes under drought stress.

Treatments	Genotypes									
	Zafar 1	Zafar 2	Giza Blanka	Espan	Makmora	Shebam 1	Giza 3	C4	C5	G853
**Plant Height (cm) Plant^−1^**										
Control	56.00 ± 0.58 ^ijk^	59.00 ± 0.58 ^ghi^	61.00 ± 0.58 ^fgh^	54.33 ± 1.20 ^jkl^	47.33 ± 1.20 ^no^	64.33 ± 1.86 ^ef^	58.00 ± 1.53 ^hij^	79.00 ± 0.58 ^c^	107.67 ± 1.45 ^a^	36.67 ± 0.88 ^st^
Mild	45.67 ± 1.76 ^op^	56.00 ± 0.58 ^ijk^	57.33 ± 1.45 ^hij^	52.00 ± 1.15 ^lm^	31.33 ± 0.33 ^uv^	62.00 ± 1.53 ^fg^	40.00 ± 1.73 ^qrs^	70.33 ± 0.33 ^d^	86.67 ± 0.88 ^b^	25.67 ± 0.88 ^w^
Moderate	37.33 ± 1.20 ^rs^	42.33 ± 1.45 ^pq^	50.00 ± 1.00 ^mn^	45.33 ± 1.76 ^op^	30.00 ± 1.53 ^uv^	39.00 ± 1.73 ^qrs^	52.67 ± 1.86 ^klm^	66.67 ± 0.88 ^e^	73.67 ± 0.33 ^d^	19.00 ± 0.58 ^x^
Severe	28.67 ± 1.67 ^vw^	38.67 ± 0.33 ^qrs^	33.67 ± 0.88 ^tu^	41.00 ± 1.73 ^qr^	21.67 ± 0.88 ^x^	28.33 ± 1.20 ^vw^	32.67 ± 1.76 ^u^	58.00 ± 0.58 ^hij^	66.67 ± 0.88 ^e^	13.67 ± 0.33 ^y^
**Shoot FW (g) Plant^−1^**										
Control	5.63 ± 0.48 ^cd^	5.10 ± 0.12 ^cdef^	4.87 ± 0.44 ^cdefgh^	5.70 ± 0.10 ^cd^	5.13 ± 0.03 ^cdef^	5.47 ± 0.78 ^cd^	5.00 ± 0.06 ^cdefg^	6.00 ± 0.31 ^c^	8.50 ± 0.23 ^a^	2.40 ± 0.06 ^nopqrs^
Mild	2.97 ± 0.87 ^klmnopqr^	3.53 ± 0.82 ^hijklmno^	4.70 ± 0.32 ^defghij^	4.53 ± 0.35 ^defghi^	4.73 ± 0.12 ^cdefgh^	3.77 ± 0.43 ^fghijklm^	4.07 ± 0.46 ^efghijk^	4.87 ± 0.12 ^cdefgh^	7.17 ± 0.15 ^b^	2.20 ± 0.06 ^opqrst^
Moderate	2.13 ± 0.52 ^pqrst^	2.03 ± 0.29 ^pqrst^	4.07 ± 0.50 ^efghijk4^	3.13 ± 0.83 ^jklmnopq^	1.80 ± 0.38 ^qrst^	3.70 ± 0.15 ^ghijklmn^	3.90 ± 0.21 ^efghijkl^	4.00 ± 0.15 ^efghijkl^	5.27 ± 0.09 ^cde^	1.43 ± 0.24 ^stu^
Severe	1.67 ± 0.35 ^rstu^	2.43 ± 0.75 ^mnopqrs^	3.27 ± 0.32 ^ijklmnoq^	2.67 ± 0.60 ^lmnopqrs^	0.90 ± 0.25 ^tu^	1.97 ± 0.49 ^pqrst^	3.17 ± 0.43 ^ijklmnop^	2.70 ± 0.06 ^lmnopqrs^	4.37 ± 0.15 ^defghij^	0.50 ± 0.06 ^u^
**Shoot DW (g) Plant^−1^**										
Control	0.76 ± 0.16 ^ab^	0.63 ± 0.06 ^abcdef^	0.70 ± 0.23 ^abc^	0.73 ± 0.07 ^ab^	0.59 ± 0.02 ^bcdefg^	0.56 ± 0.07 ^bcdefgh^	0.57 ± 0.02 ^bcdefgh^	0.56 ± 0.04 ^bcdefgh^	0.83 ± 0.03 ^a^	0.40 ± 0.01 ^ghijklm^
Mild	0.46 ± 0.02 ^efghijk^	0.40 ± 0.12 ^ghijklm^	0.67 ± 0.06 ^abcde^	0.50 ± 0.01 ^cdefghi^	0.46 ± 0.07 ^efghijkl^	0.44 ± 0.06 ^fghijklm^	0.45 ± 0.06 ^fghijklm^	0.37 ± 0.07 ^ghijklm^	0.68 ± 0.02 ^abcd^	0.29 ± 0.01 ^ijklm^
Moderate	0.41 ± 0.01 ^fghijklm^	0.30 ± 0.09 ^ijklm^	0.46 ± 0.05 ^efghijkl^	0.45 ± 0.05 ^fghijklm^	0.36 ± 0.02 ^hijklm^	0.42 ± 0.06 ^fghijklm^	0.47 ± 0.03 ^defghij^	0.25 ± 0.00 ^klm^	0.50 ± 0.01 ^cdefghi^	0.24 ± 0.07 ^lm^
Severe	0.30 ± 0.01 ^ijklm^	0.23 ± 0.07 ^m^	0.41 ± 0.03 ^ghijklm^	0.42 ± 0.06 ^fghi^	0.24 ± 0.05 ^klm^	0.28 ± 0.03 ^ijklm^	0.28 ± 0.03 ^ijklm^	0.25 ± 0.01 ^jklm^	0.35 ± 0.01 ^hijklm^	0.01 ± 0.00 ^n^
**Area (cm^2^) Leaf^−1^**										
Control	20.00 ± 0.58 ^c^	13.34 ± 0.69 ^fgh^	12.25 ± 0.89 ^ghij^	15.60 ± 0.74 ^de^	14.79 ± 0.41 ^ef^	14.91 ± 0.55 ^ef^	12.59 ± 0.47 ^ghi^	7.63 ± 0.15 ^opqrs^	27.33 ± 0.88 ^a^	8.53 ± 0.34 ^nopqr^
Mild	17.00 ± 0.58 ^d^	11.82 ± 0.32 ^hij^	12.17 ± 0.58 ^ghij^	13.91 ± 0.36 ^efg^	11.38 ± 0.35 ^ijk^	10.94 ± 0.88 ^ijkl^	10.79 ± 0.76 ^ijklm^	6.56 ± 0.53 ^rstu^	23.37 ± 0.32 ^b^	7.17 ± 0.18 ^pqrst^
Moderate	11.33 ± 0.88 ^ijk^	8.73 ± 0.44 ^nopq^	9.77 ± 0.79 ^klmn^	9.35 ± 0.34 ^lmno^	8.51 ± 0.71 ^nopqr^	9.00 ± 0.58 ^mnop^	9.78 ± 0.53 ^klmn^	6.17 ± 0.15 ^stu^	20.00 ± 0.58 ^c^	5.98 ± 0.39 ^stu^
Severe	10.63 ± 0.55 ^jklm^	7.27 ± 0.92 ^pqrst^	7.41 ± 0.89 ^opqrst^	5.65 ± 0.42 ^tuv^	7.00 ± 0.47 ^qrst^	7.15 ± 0.69 ^p^	7.92 ± 0.56 ^nopqrs^	5.03 ± 0.93 ^uv^	16.76 ± 0.39 ^d^	4.10 ± 0.32 ^v^

Means followed by a similar letter within a column for each parameter are not significantly different at the 0.05 level of probability by Duncan’s Multiple-Range Test.

**Table 2:** Effect of drought stress on leaf relative water content (RWC), proline (Pro) content and total chlorophyll (Total *Chlo*) content in faba bean genotypes.

Treatments	Genotypes									
	Zafar 1	Zafar 2	Giza Blanka	Espan	Makamora	Shebam 1	Giza 3	C4	C5	G853
**RWC %**										
Control	75.19 ± 0.36 ^de^	78.15 ± 0.28 ^b^	73.23 ± 0.29 ^f^	71.05 ± 0.69 ^g^	73.03 ± 0.33 ^f^	76.51 ± 0.76 ^cd^	69.14 ± 0.10 ^h^	76.89 ± 0.44 ^bc^	80.84 ± 0.33 ^a^	62.71 ± 0.55 ^n^
Mild	64.89 ± 0.50 ^lm^	70.84 ± 0.53 ^g^	66.14 ± 0.89 ^ijkl^	69.24 ± 0.44 ^h^	71.24 ± 0.58 ^g^	64.14 ± 0.66 ^m^	65.20 ± 0.50 ^klm^	65.09 ± 0.38 ^klm^	74.03 ± 0.13 ^ef^	53.89 ± 0.05 ^r^
Moderate	66.92 ± 0.33 ^ij^	66.26 ± 0.37 ^ijkl^	60.96 ± 0.70 ^o^	66.51 ± 0.59 ^ijk^	55.71 ± 0.60 ^q^	57.18 ± 0.69 ^p^	60.92 ± 0.30 ^o^	60.20 ± 0.55 ^o^	68.12 ± 0.23 ^hi^	50.13 ± 0.18 ^tu^
Severe	34.48 ± 0.46 ^z^	44.39 ± 0.30 ^w^	49.19 ± 0.11 ^uv^	41.79 ± 0.20 ^x^	45.26 ± 0.45 ^w^	48.15 ± 0.11 ^v^	50.87 ± 0.26 ^st^	38.23 ± 0.94 ^y^	52.09 ± 0.14 ^s^	25.82 ± 0.65 ^z^
**Pro (μg^−1^·FW)**										
Control	0.91 ± 0.01 ^opq^	1.30 ± 0.02 ^hijkl^	0.90 ± 0.06 ^pq^	1.05 ± 0.02 ^lmnopq^	0.56 ± 0.03 ^r^	0.89 ± 0.07 ^pq^	0.91 ± 0.03 ^opq^	1.21 ± 0.03 i^jklmn^	1.34 ± 0.01 ^hijk^	0.49 ± 0.02 ^r^
Mild	1.11 ± 0.01 ^klmnopq^	1.85 ± 0.02 ^f^	1.17 ± 0.03 ^ijklmnop^	1.21 ± 0.09 ^e^	0.99 ± 0.01 ^mnopq^	0.91 ± 0.05 ^opq^	1.10 ± 0.05 ^klmnopq^	1.45 ± 0.01 ^hi^	2.42 ± 0.02 ^d^	0.51 ± 0.03 ^r^
Moderate	1.78 ± 0.03 ^fg^	2.28 ± 0.06 ^de^	1.24 ± 0.01 ^ijklm^	2.11 ± 0.05 ^ijklmn^	1.19 ± 0.02 ^ijklmno^	1.19 ± 0.01 ^ijklmno^	1.21 ± 0.07 ^ijklmn^	1.55 ± 0.01 ^gh^	2.82 ± 0.01 ^c^	0.84 ± 0.01 ^fg^
Severe	2.29 ± 0.11 ^de^	3.11 ± 0.42 ^b^	1.43 ± 0.02 ^hijk^	2.53 ± 0.03 ^d^	1.82 ± 0.08 ^f^	1.52 ± 0.17 ^h^	1.16 ± 0.01 ^jklmnop^	2.82 ± 0.03 ^c^	3.65 ± 0.06 ^a^	0.94 ± 0.01 ^nopq^
**Total *Chl* (mg·g^−1^·FW)**										
Control	40.47 ± 0.53 ^cde^	40.07 ± 0.74 ^cde^	40.87 ± 0.59 ^cd^	41.50 ± 0.42 ^bcd^	38.77 ± 0.67 ^efg^	37.43 ± 0.76 ^gh^	38.00 ± 0.06 ^f^	41.97 ± 0.24 ^bcd^	46.77 ± 0.59 ^a^	35.00 ± 0.58 ^j^
Mild	37.27 ± 0.62 ^ghi^	38.00 ± 0.85 ^fg^	35.40 ± 0.23 ^ij^	35.37 ± 0.35 ^ij^	38.00 ± 0.58 ^fg^	32.77 ± 0.19 ^kl^	37.67 ± 0.88 ^fg^	39.67 ± 0.39 ^def^	42.80 ± 0.23 ^b^	27.00 ± 0.58 ^p^
Moderate	35.60 ± 0.60 ^hij^	32.33 ± 0.88 ^l^	34.43 ± 0.70 ^jk^	32.40 ± 0.83 ^l^	32.83 ± 0.44 ^kl^	29.27 ± 0.43 ^no^	32.57 ± 0.78 ^kl^	35.67 ± 0.93 ^hij^	37.80 ± 0.46 ^fg^	23.67 ± 0.88 ^q^
Severe	32.00 ± 0.58 ^l^	26.83 ± 0.44 ^p^	27.80 ± 0.20 ^op^	29.90 ± 0.90 ^mn^	31.03 ± 0.98 ^lmn^	29.27 ± 0.73 ^no^	32.23 ± 0.62 ^l^	29.73 ± 0.37 ^mn^	31.43 ± 0.43l ^m^	18.67 ± 0.88 ^r^

Means followed by a similar letter within a column for each parameter are not significantly different at the 0.05 level of probability by Duncan's Multiple-Range Test.

**Table 3:** Effect of drought stress on electrolyte leakage and content of malondialdehyde (MDA) and hydrogen peroxide (H_2_O_2_) in faba bean genotypes.

Treatments	Genotypes									
	Zafar 1	Zafar 2	Giza Blanka	Espan	Makamora	Shebam 1	Giza 3	C4	C5	G853
**Electrolyte Leakage (%)**										
Control	32.67 ± 1.45 ^n^	37.00 ± 1.15 ^n^	35.33 ± 1.45 ^n^	36.00 ± 1.53 ^n^	37.67 ± 1.45 ^n^	43.33 ± 1.76 ^m^	37.33 ± 1.20 ^n^	37.67 ± 1.45 ^n^	35.00 ± 0.58 ^n^	47.00 ± 1.15 ^klm^
Mild	45.00 ± 2.65 ^m^	46.67 ± 1.20 ^klm^	46.33 ± 0.88 ^lm^	48.00 ± 1.53 ^klm^	55.33 ± 1.20 ^ij^	55.33 ± 1.76 ^ij^	51.33 ± 1.86 ^jkl^	45.33 ± 1.45 ^m^	45.00 ± 2.65 ^m^	57.67 ± 1.45 ^hi^
Moderate	55.67 ± 2.03 ^ij^	55.33 ± 2.03 ^ij^	53.67 ± 2.33 ^ij^	64.00 ± 2.65 ^efg^	66.00 ± 1.73 ^defg^	66.00 ± 2.08 ^defg^	57.67 ± 0.88 ^hi^	61.67 ± 1.20 ^gh^	51.67 ± 2.03 ^jk^	66.67 ± 1.67 ^defg^
Severe	66.00 ± 1.53 ^defg^	67.00 ± 1.15 ^def^	69.00 ± 0.58 ^cde^	70.33 ± 0.88 ^cd^	76.33 ± 2.19 ^ab^	72.33 ± 1.20 ^bc^	67.67 ± 1.33 ^cde^	76.00 ± 1.53 ^ab^	62.33 ± 1.45 ^fgh^	77.67 ± 1.45 ^a^
**MDA Content (nmol·g^−1^·FW)**										
Control	27.33 ± 1.20 ^n^	27.00 ± 1.15 ^n^	24.67 ± 2.03 ^n^	26.00 ± 1.53^n^	27.67 ± 1.45 ^n^	33.33 ± 1.76 ^m^	25.00 ± 0.58 ^n^	27.67 ± 1.45 ^n^	22.33 ± 1.45 ^n^	37.00 ± 1.15 ^lm^
Mild	41.33 ± 1.86 ^kl^	36.67 ± 1.20 ^lm^	36.33 ± 0.88 ^lm^	38.00 ± 1.53 ^lm^	45.33 ± 1.20 ^jk^	45.33 ± 1.76 ^jk^	35.00 ± 2.65 ^m^	35.33 ± 1.45 ^m^	35.00 ± 2.65 ^m^	47.67 ± 1.45 ^ij^
Moderate	45.67 ± 2.03 ^jk^	45.33 ± 2.03 ^jk^	51.67 ± 1.20 ^hi^	54.00 ± 2.65 ^fgh^	56.00 ± 1.73 ^efgh^	56.00 ± 2.08 ^efgh^	47.67 ± 0.88 ^ij^	43.67 ± 2.33 ^jk^	45.00 ± 1.73 ^jk^	57.67 ± 1.45 ^defg^
Severe	55.33 ± 2.03 ^efgh^	60.33 ± 0.88 ^cde^	58.33 ± 1.20 ^def^	62.33 ± 1.20 ^bcd^	64.33 ± 2.60 ^abc^	55.33 ± 1.86 ^efgh^	57.67 ± 1.33 ^defg^	67.00 ± 1.53 ^ab^	52.33 ± 1.45 ^ghi^	67.67 ± 1.45 ^a^
**H_2_O_2_ Content (mμ·mol g^−1^·leaf·FW)**										
Control	15.00 ± 0.58 ^q^	18.67 ± 0.88 ^o^	16.00 ± 0.58 ^pq^	18.00 ± 1.15 ^op^	18.00 ± 0.58 ^op^	18.67 ± 0.88 ^o^	20.00 ± 0.58 ^no^	19.00 ± 0.58 ^o^	15.00 ± 0.58 ^q^	18.67 ± 0.33 ^o^
Mild	23.33 ± 0.88 ^lm^	24.67 ± 0.67 ^kl^	22.00 ± 1.15 ^mn^	24.00 ± 0.58 ^lm^	20.33 ± 0.88 ^no^	26.00 ± 0.58 ^jkl^	27.00 ± 1.15 ^jk^	25.00 ± 0.58 ^kl^	19.00 ± 0.58 ^o^	25.33 ± 0.88 ^kl^
Moderate	29.67 ± 0.88 ^hi^	28.33 ± 0.8 8 ^ij^	29.67 ± 0.88 ^hi^	30.00 ± 1.15 ^hi^	26.00 ± 1.15 ^jkl^	31.00 ± 0.58 ^gh^	33.33 ± 0.88 ^efg^	27.00 ± 0.58 ^jk^	23.33 ± 0.88 ^lm^	32.00 ± 1.15 ^fgh^
Severe	34.67 ± 0.88 ^cde^	33.67 ± 0.88 ^def^	35.00 ± 0.58 ^cde^	37.67 ± 0.88 ^b^	31.33 ± 0.88 ^fgh^	36.00 ± 0.58 ^bcd^	37.00 ± 0.58 ^bc^	31.67 ± 0.88 ^fgh^	28.33 ± 0.88 ^ij^	43.67 ± 0.88 ^a^

Means followed by a similar letter within a column for each parameter are not significantly different at the 0.05 level of probability by Duncan's Multiple-Range Test.

**Table 4:** Effect of drought stress on the activity of catalase (CAT), peroxidase (POD) and superoxide dismutase (SOD) in faba bean genotypes.

Treatments	Genotypes									
	Zafar 1	Zafar 2	Giza Blanka	Espan	Makamora	Shebam 1	Giza 3	C4	C5	G853
**CAT Activity (units·mg ^−1^·protein·min ^−1^)**										
Control	130.3 ± 0.88 ^r^	142.7 ± 1.45 ^pq^	143.0 ± 1.15 ^pq^	143.3 ± 2.60 ^pq^	165.0 ± 1.73 ^lm^	155.0 ± 1.73 ^no^	191.0 ± 2.08 ^fg^	149.7 ± 1.45 ^nop^	180.0 ± 3.61 ^hi^	139.3 ± 0.88 ^q^
Mild	148.7 ± 1.76 ^op^	178.0 ± 1.73 ^hi^	156.0 ± 2.08 ^n^	167.7 ± 3.76 ^klm^	172.3 ± 1.20 ^jk^	171.0 ± 2.08 ^jk^	197.0 ± 2.31 ^f^	181.3 ± 1.86 ^hi^	206.0 ± 3.06 ^e^	154.7 ± 2.60 ^no^
Moderate	164.7 ± 1.76 ^lm^	195.0 ± 1.73 ^f^	163.0 ± 2.08 ^m^	178.0 ± 1.53 ^hi^	182.3 ± 1.45 ^h^	184.0 ± 1.53 ^h^	194.0 ± 5.13 ^f^	209.0 ± 3.21 ^e^	222.7 ± 1.45 ^cd^	163.3 ± 2.03 ^m^
Severe	247.7 ± 1.45 ^a^	204.3 ± 2.33 ^e^	185.3 ± 2.03 ^gh^	216.3 ± 4.10 ^d^	194.0 ± 2.31 ^f^	223.7 ± 2.73 ^c^	220.3 ± 1.20 ^cd^	237.3 ± 2.33 ^b^	242.7 ± 1.76 ^ab^	175.0 ± 1.73 ^ij^
**POD Activity (unit·min^−1^·g^−1^·FW)**										
Control	20.50 ± 0.23 ^kl^	20.33 ± 0.32 ^lm^	13.70 ± 0.12 ^q^	16.27 ± 0.27 ^o^	19.83 ± 0.15 ^lm^	11.27 ± 0.27 ^r^	16.43 ± 0.24 ^o^	9.87 ± 0.47 ^s^	21.33 ± 0.23 ^jk^	13.63 ± 0.18 ^q^
Mild	23.10 ± 0.31 ^hi^	23.30 ± 0.31 ^hi^	16.30 ± 0.23 °	19.50 ± 0.17 ^m^	23.50 ± 0.21 ^gh^	13.90 ± 0.23 ^q^	20.40 ± 0.32 ^lm^	11.93 ± 0.47 ^r^	24.67 ± 0.12 ^ef^	15.70 ± 0.26 ^op^
Moderate	25.37 ± 0.18 ^e^	25.53 ± 0.23 ^e^	19.77 ± 0.47 ^lm^	21.60 ± 0.15 ^j^	26.57 ± 0.18 ^d^	18.50 ± 0.23 ^n^	24.30 ± 0.26 ^fg^	15.30 ± 0.26 ^p^	27.23 ± 0.24 ^bcd^	20.40 ± 0.32 ^lm^
Severe	27.50 ± 0.23 ^bc^	26.67 ± 0.30 ^cd^	24.47 ± 0.85 ^f^	24.33 ± 0.32 ^fg^	28.10 ± 0.12 ^b^	20.53 ± 0.23 ^kl^	26.53 ± 0.23 ^d^	18.30 ± 0.29 ^n^	29.50 ± 0.21 ^a^	22.47 ± 0.20 ^i^
**SOD Activity (units·mg^−1^·protein·min^−1^)**										
Control	9.33 ± 0.33 ^rst^	10.00 ± 0.58 ^qrs^	11.00 ± 0.58 ^pqr^	6.00 ± 0.58 ^v^	8.00 ± 0.58 ^tu^	7.00 ± 0.58 ^uv^	8.67 ± 0.33 ^stu^	7.33 ± 0.33 ^uv^	11.33 ± 0.33 ^pq^	14.00 ± 0.58 ^mn^
Mild	15.33 ± 0.67 ^lm^	14.00 ± 0.58 ^mn^	14.67 ± 0.67 ^lmn^	14.00 ± 0.58 ^mn^	12.00 ± 0.58 ^op^	9.67 ± 0.33 ^qrst^	11.33 ± 0.33 ^pq^	13.33 ± 0.33 ^no^	17.67 ± 0.33 ^ijk^	15.67 ± 0.67 ^lm^
Moderate	20.00 ± 0.58 ^efg^	19.00 ± 0.58 ^ghi^	20.00 ± 0.58 ^efg^	18.00 ± 0.58 ^hij^	17.67 ± 0.88 ^ijk^	14.00 ± 0.58 ^mn^	16.00 ± 1.15 ^kl^	16.33 ± 0.33 ^jkl^	21.33 ± 0.88 ^def^	18.67 ± 0.33 ^ghi^
Severe	25.00 ± 0.58 ^b^	24.00 ± 0.58 ^bc^	25.00 ± 0.58 ^b^	22.67 ± 0.88 ^cd^	21.67 ± 0.88 ^de^	24.33 ± 0.33 ^bc^	21.00 ± 0.58 ^def^	19.67 ± 0.33 ^fgh^	28.00 ± 0.58 ^a^	20.33 ± 0.33 ^efg^

Means followed by a similar letter within a column for each parameter are not significantly different at the 0.05 level of probability by Duncan's Multiple-Range Test.

## 3. Experimental Section

### 3.1. Plant and Treatment

Seeds of 10 improved genotypes of *Vicia faba* L. were obtained from different geographical origins. Seeds of genotypes Zafar 1, Zafar 2 and Shebam from the General organization for Agriculture Research, Yemen, genotypes Makamora and Espan from the local market of Riyadh and genotypes Giza Blanka, Giza 3, C4, C5 and G853 from Agriculture Research Center, Egypt. The experiment was conducted in a growth chamber (temperature 25 ± 3 °C, relative humidity 50%–60%, light 250 μmol of photons m^−2^·s^−1^ on a 16/8**-**h light/dark cycle). Seeds were grown in pots containing a mixture of sand and peat (1:1). Drought stress was initiated when seedlings attained 2–3 true leaves. Drought stress treatments were imposed by withholding water. The details of the drought stress treatments were as follows: (i) Control (UN): Normal irrigation (irrigated alternate day); (ii) Mild stress (D1): Irrigation after 3 days of control plants irrigation; (iii) Moderate stress (D2): Irrigation after 6 days of control plants irrigation; and (iv) Severe stress (D3): Irrigation after 9 days of control plants irrigation. The experimental pots were arranged in a simple randomized design with five replicates per treatment. Before sowing, seeds of all genotypes were surface sterilized with 1% sodium hypochlorite for 10 min, then were vigorously rinsed with double distilled water (DDW) and sown in sand+peat-filled pots supplied with Raukura’s nutrient solution [34]. The salts used to make up the nutrient solution are as follows: Macronutrient stock solution A (g·L^−1^) Mg(NO_3_)_2_·6H_2_O, 4.94; Ca(NO_3_)_2_·4H_2_O, 16.78; NH_4_NO_3_, 8.48; KNO_3_, 2.28. Macronutrient stock solution B (g·L^−1^) KH_2_PO_4_, 2.67; K_2_HPO_4_, 1.64; K_2_SO_4_, 6.62; Na_2_SO_4_, 0.60; NaCl, 0.33. Micronutrient supplement (mg·L^−1^) H_3_BO_3_, 128.80; CuCl_2_·2H_2_O, 4.84; MnCl_2_·4H_2_O, 81.10; (NH_4_)6Mo_7_O_24_·4H_2_O, 0.83; ZnCl_2_, 23.45; ferric citrate pentahydrate, 809.84. The dilute solution which was applied to the plants was prepared by mixing 200 mL of each of the macronutrient stock solution with 100 mL of the micronutrient supplement and was diluted to 4.5 L with DDW.

Sampling was done after 50 days of sowing. The growth performance of faba bean plants was assessed in terms of plant height (PH) plant^−1^, fresh weight (FW) and dry weight (DW) plant^−1^ and area (A) leaf^−1^.

The leaf area was measured directly using Leaf Area Meter (Model LI-3050A, LI-COR Inc, Lincon, NE, USA). The area of three leaves (upper, middle, and lower) of each plant of the sample (consisting of five plants) was determined.

### 3.2. Determination of Physio-Biochemical Characteristics

#### 3.2.1. Leaf Relative Water Content

Leaf RWC (%) was determined using the methods of Gulen and Eris [35]. Leaf discs of 1.5 cm diameter were taken from the fully expanded and uniform leaves of each of the three plants (replicates) per treatment. First, the FW was recorded, and then samples were placed in a petri dish having distilled water for 4 h. Turgid weight (TW) was then recorded, and the leaf samples were placed in an incubator at 70 °C for 24 h, to determine the dry weight. Leaf RWC % was calculated by:

RWC (%) = [(FW − DW)/(TW − DW)] × 100

#### 3.2.2. Total Chlorophyll Concentration

The youngest fully expanded leaves were subjected to extraction using 80% acetone, and the absorbance was measured using UV-vis Spectrophotometer (SPEKOL 1500; Analytik Jena AG, Jena, Germany) at 663 and 645 nm. The total chlorophyll content was determined by using Arnonʼs formula [36].

#### 3.2.3. Proline Concentration

The proline concentration was determined spectrophotometrically using the ninhydrin method of Bates *et al.* [37]. First, fresh leaf samples were homogenized in 3% sulfosalicylic acid, followed by the addition of 2 mL each of ninhydrin and glacial acetic acid, after which the samples were heated to 100 °C. The mixture was then extracted with toluene, and the free toluene was quantified at 520 nm.

#### 3.2.4. MDA Concentration

The MDA content was determined according to the method of Heath and Packer [38]. Leaf samples were weighed, and homogenates containing 10% trichloroacetic acid and 0.65% 2-thiobarbituric acid were heated at 95 °C for 60 min, then cooled to room temperature, and centrifuged at 10,000× *g* for 10 min. The absorbance of the supernatant was read at 532 and 600 nm against a reagent blank.

#### 3.2.5. Electrolyte Leakage

Electrolyte leakage was used to assess membrane permeability in accordance with Lutts *et al.* [39]. Samples were washed 3 times with double-distilled water to remove surface contamination, and leaf discs were cut from young leaves and placed in sealed vials containing 10 mL of DDW, followed by incubation on a rotary shaker for 24 h, after which the electrical conductivity of the solution (EC1) was determined. Then, the samples were autoclaved at 120 °C for 20 min, and the electrical conductivity was measured again (EC2) after the solution was cooled to room temperature. The electrolyte leakage was defined as EC1/EC2 × 100 and expressed as percentage.

#### 3.2.6. Hydrogen Peroxide (H_2_O_2_)

It was measured as described by Velikova *et al.* [40]. Fresh leaf samples (0.5 g) were homogenized in 5 mL of 0.1% (*w*/*v*) TCA. The homogenate was centrifuged at 12,000 rpm for 15 min and the supernatant was added to 10 mM potassium phosphate buffer (pH 7.0) and 1 M potassium iodide. The absorbance of the supernatant was recorded at 390 nm. The content of H_2_O_2_ was calculated by comparison with a standard calibration curve plotted using known concentrations of H_2_O_2_.

### 3.3. Determination of Antioxidant Enzymes’ Activity

To determine the activities of antioxidant enzymes, a crude enzyme extract was prepared by homogenizing 500 mg of leaf tissue in extraction buffer (0.5% Triton X-100 and 1% polyvinylpyrrolidone in 100 mM potassium phosphate buffer, pH 7.0) using a chilled mortar and pestle. The homogenate was then centrifuged at 15,000× *g* for 20 min at 4 °C, and the supernatant was used for the enzymatic assays described below. All enzyme activities were expressed as milligram of protein per minute.

We applied the method of Chance and Maehly [41] to determine POD (EC 1.11.1.7) activity using 5 mL of enzyme reaction solution containing phosphate buffer (pH 6.8), 50 M pyrogallol, 50 mM H_2_O_2_, and 1 mL of the enzyme extract diluted 20 times. The assay mixture was incubated for 5 min at 25 °C, and the reaction was terminated by the addition of 0.5 mL of 5% (*v*/*v*) H_2_SO_4_. Purpurogallin production was measured spectrophotometrically at 420 nm. One unit of POD activity was considered the amount of purpurogallin formed per milligram of protein per minute.

The method of Aebi [42] was used to measure CAT (EC 1.11.1.6) activity. The decomposition of H_2_O_2_ was measured as the decrease in absorbance at 240 nm. In this assay, 50 mM phosphate buffer (pH 7.8) and 10 mM H_2_O_2_ were used in the reaction solution.

Activity of SOD (EC 1.15.1.1) was determined based on the inhibition of nitro blue tetrazolium (NBT) photoreduction according to the method of Giannopolitis and Ries [43]. The reaction solution (3 mL) contained 50 mM NBT, 1.3 mM riboflavin, 13 mM methionine, 75 µM ethylenediamine tetraacetic acid (EDTA), 50 mM phosphate buffer (pH 7.8), and 20 to 50 mL of enzyme extract. The reaction solution was irradiated under fluorescent light at 75 µM·m**^−^**^2^·s**^−^**^1^ for 15 min. The absorbance at 560 nm was read against a blank (non-irradiated reaction solution). One unit of SOD activity was defined as the amount of enzyme that inhibited 50% of NBT photoreduction.

### 3.4. Statistical Analysis

The data were expressed as the mean ± standard error and were analyzed statistically using IBM SPSS Ver.22 statistical software (IBM Corporation and Others, Armonk, NY, USA). The means were compared statistically using Duncan’s multiple-range test at the level of *p* < 0.05.

## 4. Conclusions

All morphological, physiological and biochemical characteristics of 10 genotypes of faba bean reduced under drought stress. We observed all genotypes of faba bean behaved differently under water stress. In the present study, we found some of the genotypes were tolerant (“C5” and “Zafar 1”), mild tolerant (“Giza 3”, “Zafar 2”, “Giza Blanka”, “Espan”, “Shebam 1” and “Makamora”) and sensitive (“G853” and “C4”) to water stress. However, genotypes “C5” and “Zafar 1” performed better by improving RWC and accumulation of Pro. Also, tolerant genotypes had a better ability to reduce oxidative damage by increasing activity of CAT, POD and SOD. For further study, theses genotypes can be used to uncover molecular mechanism(s) involved in building the tolerance of faba bean plants to drought stress.
